# Match injuries in amateur Rugby Union: a prospective cohort study - FICS Biennial Symposium Second Prize Research Award

**DOI:** 10.1186/s12998-016-0098-7

**Published:** 2016-06-01

**Authors:** Michael S. Swain, Reidar P. Lystad, Nicholas Henschke, Christopher G. Maher, Steven J. Kamper

**Affiliations:** The George Institute for Global Health, Sydney Medical School, University of Sydney, GPO Box 5389, Sydney, NSW 2001 Australia; Department of Chiropractic, Faculty of Science and Engineering, Macquarie University, Sydney, Australia; School of Medical and Applied Sciences, Central Queensland University, Sydney, Australia; Institute of Public Health, University of Heidelberg, Heidelberg, Germany

**Keywords:** Sports, Football, Rugby Union, Athletic injuries, Epidemiology

## Abstract

**Background:**

The majority of Rugby Union (rugby) players participate at the amateur level. Knowledge of player characteristics and injury risks is predominantly ascertained from studies on professional or junior athletes in rugby. The objectives of the current study are to: (1) describe the health-related quality of life (HRQoL) and physical characteristics of a cohort of amateur rugby players; (2) describe the incidence, severity and mechanism of match injuries in amateur rugby, and; (3) explore factors associated with rates of match injury in this population.

**Methods:**

Participants (*n* = 125) from one amateur men’s rugby club were followed in a one-season (2012) prospective cohort study. Match injury and match time exposure data were collected. A participant match exposure log was maintained. Baseline variables collected include: participant’s age, playing experience, position of play, the SF-36v2 health survey, height and weight. Injury incidence rates (IIRs) per 1000 match-hours exposure were calculated. Injury sub-groups were compared by calculating rate ratios of two IIRs. Poisson mixed-effects generalised linear modelling was used to explore relationships between IIRs and baseline predictors.

**Results:**

A total of 129 injuries occurred during a combined period of 2465 match-hours of exposure. The overall IIR was 52.3 (43.7–62.2) /1000 match-hours exposure. Moderate-severe injuries (>1 week time-loss from play) comprised 36 % of all injuries. Tackling was the most common mechanism of injury, the head/face was the most common body region of injury and sprain/ligament injuries were the most common injury type. Fewer years of rugby participation, lower BMI and lower SF-36v2 mental component summary score were associated with higher IIR in amateur rugby. Age, player position i.e., backs versus forwards and SF-36v2 physical component summary score were not associated with injury incidence.

**Conclusion:**

Amateur rugby players report similar HRQoL as the general population. We found amateur players had a higher rate of injury and lower injury severity than previous amateur studies, but location, type, and mechanism were similar. In this study pre-season HRQoL and BMI were weakly associated with higher injury rate when controlling for other factors; a finding that should be interpreted with caution and clarified with future research.

**Electronic supplementary material:**

The online version of this article (doi:10.1186/s12998-016-0098-7) contains supplementary material, which is available to authorized users.

## Background

Rugby Union (rugby) is a contact sport that is popular worldwide. Well known health benefits of sport and physical activity include improved cardiorespiratory and muscular fitness, bone health [1, 2] and reduced risk of non-communicable diseases such as obesity and depression [3]. Vigorous-intensity physical activity can further challenge aerobic fitness and muscle strength which is thought to provide additional improvements to health and wellbeing [4]. Rugby has been promoted in at least one public health campaign [5]. The stated benefits of rugby were not limited to physical health, but also included team participation, social interaction, communication skills and self-discipline.

The health benefits of sport participation should be weighed against inherent risks, which mostly exist as sport-related musculoskeletal injury. The risk of incurring an injury in rugby appears to be higher than in many other sports but comparable with other contact sports [6, 7] such as wrestling. Lee et al., [8] found approximately 10 % of participants stopped playing rugby completely as a result of injury. More than one-third of players report temporary or significant effects on education, employment, family life, or health and general fitness. For example, restriction or cessation of sporting activity, and continuing pain or stiffness [8]. Hence, the majority of rugby participants that incur an injury will experience minor health-related consequences. The need to put player’s welfare first along with the rare occurrence of catastrophic injuries (e.g., spinal cord injury) [9] necessitates a greater understanding of both player’s health and injury risks.

Factors that are thought to increase the risk of incurring a match injury in rugby include: increasing age [10] and more senior grade of play [11], high strenuous physical activity per week [10] and higher pre-season training attendance [12], playing while injured [10, 12], carrying an injury from the previous season [11, 12], and foul-play [10]. These reported increases in risk are typically small and are inconsistent between studies. While there has been some exploration on the influence of rugby injuries on players’ subsequent health and lifestyle in amateur rugby, it is currently unclear whether health and wellbeing plays a role in the aetiology of sports injuries in amateur athletes. The New Zealand Rugby Injury and Performance Project undertaken by Quarrie et al., evaluated self-reported health status (response options: very good, good, not too good) and psychological wellbeing (measured using the General Health Questionnaire) as risk factors for injury in rugby. They found rugby players who reported negative health and psychological wellbeing did not differ significantly in the rate of injury compared to participants who reported positive health and wellbeing outcomes. Conversely, a large population-based study from New Zealand found a linear relationship between poorer health status and increasing injury risk, with the majority of injuries being sports-related [13].

Worldwide rugby participation continues to rise [14]. In Australia, recent statistics indicate that participation is slightly higher in junior age-groups [15] (juniors 50,000 vs. seniors 41,000) and the vast majority of rugby players participate at the amateur level [16]. Several prospective studies have investigated the epidemiology of sports injury in rugby [17, 18]; however these have largely consisted of cohorts of elite or junior athletes. There is a need to further explore health and injury in amateur rugby to better inform athletes of the potential benefits and harms as well as minimise risks associated with amateur participation.

The objectives of the current study are to: (1) describe health-related quality of life (HRQoL) and physical characteristics of a cohort of amateur rugby players; (2) describe the incidence, severity and mechanism of match injuries in amateur rugby, and; (3) explore factors associated with rates of injury in this population.

## Methods

Ethics approval was received from the Ethics Review Committee (Human Research), Macquarie University, Sydney, Australia (reference number: 5201100183). Rugby players gave written consent to participate in the study. The study design was informed by the consensus statement on injury definitions and data collection procedures for studies of injuries in rugby [19].

### Study population and sample

A prospective cohort study was conducted during the 2012 rugby season. Participants were recruited pre-season (March 2012) from one Australian amateur rugby club located in Sydney’s northern suburbs. All participants were registered male amateur club players, aged 18 years and older.

### Data collection

At recruitment, participants completed a self-reported questionnaire and underwent a physical assessment. The questionnaire gathered information regarding participant’s age, playing experience, position of play and the SF-36v2 health-related quality of life survey. The physical assessment consisted of free-standing height (cm) and body mass (kg) measurements using a Seca digital column scale.

Injury was defined according to the Rugby Union consensus statement [20] as *any physical complaint, which was caused by a transfer of energy that exceeded the body’s ability to maintain its structural and/or functional integrity, that was sustained by a player during a rugby match or rugby training, irrespective of the need for medical attention or time-loss from rugby activities.* In this study, only injuries that occurred during competition matches were recorded. A recurrent injury is defined as *an injury of the same type and at the same site as an index injury and which occurs after a player’s return to full participation from the index injury*. Injury severity was reported as *the total number of days that have elapsed from the date of injury to the date of the player’s return to full participation in team training or available for match selection* and injuries were categorised as slight (0–1 day), minimal (2–3 days), mild (4–7 days), moderate (8–28 days) and severe (>28 days). Actual time-loss was determined by conducting in-person or telephone follow-up of injured athletes. Finally, a non-fatal catastrophic injury was defined as *a brain or spinal cord injury that results in permanent (>12 months) severe functional disability*.

Match injuries were recorded by trained research assistants who were aligned with the rugby club’s sports medicine personnel (a registered chiropractor). The research assistants attended all matches and tracked injured participants throughout the season.

Injury data were collected using the Injury Report Form for Rugby Union as outlined in the Rugby Union data collection consensus document [19]. Player grade was recorded at time of injury. Players were graded from 1st to 4th, with first grade players considered the highest level of play. In addition, a separate age-restricted grade; less than 21 years (Colts) was also followed. The time within the match that injury occurred was recorded as 1st quarter, 2nd quarter, 3rd quarter, 4th quarter or extra time. The position of play at the time of the injury was recorded, as was the injured body part, side of body and type of injury. The diagnosis of injury as identified by the club’s sports medical personnel was coded according to the Orchard Sports Injury Classification System version 10.1 (OSICS-10.1) [21]. Details regarding whether their injury was recurrent, was caused by either overuse (repetitive strain) or single trauma and the type of contact were recorded. Details pertaining to whether the injury was a result of a violation of the laws of the game were recorded. The season for this cohort began on the 14th April and ended on the 25th August 2012, consisting of 17 rounds of competition.

Individual participant match-exposures were recorded over the course of the season. Each match typically lasted 60 min and exposure logs for each member were kept to the nearest half game, match-exposures were used to calculate the total match-hour exposure (MHE) during the 2012 season. Training exposure and training injuries were not recorded.

### Data analysis

Injury incidence rates (IIRs) per 1000 h of match exposure were calculated by dividing the number of recorded injuries by the number of hours of match-play, multiplied by 1000. Player health-related quality of life (physical component summary [PCS] scores and mental component summary [MCS] scores), and physical characteristics (height, weight and BMI) were stratified by age-group and position of play, and expressed as means with standard deviations. SF-36v2 norm-based scores were calculated using gender/age-group matched Australian population data from The South Australian Health Omnibus Survey (SAHOS), 2008 [22]. PCS and MCS were calculated using Australian factor score coefficient weights [23]. In norm-based scoring, each scale is scored to have the same average (50) and the same standard deviation (10), meaning each point equals one-tenth of a standard deviation [24].

Regarding injuries, sub-groups were compared by calculating rate ratios (RR) of two IIRs. Ninety-five percent confidence intervals (95 % CIs) were calculated using standard formulae for Poisson rates [25]. The 95 % CIs for RRs were used to determine whether two rates or proportions differed significantly from one another, that is, two IIRs were deemed statistically different from one another if the 95 % CI for their RR did not include the number 1.

Poisson mixed-effects generalised linear modelling was also used to explore the multivariate relationships between IIRs and potential predictors as hypothesised a priori (age, participation years, playing position (forwards versus backs), BMI and SF-36v2 summary scores). The mixed-effects model used a random intercept for each athlete to account for the correlation induced by multiple observations of the same person. All analyses were performed using the statistical software R version 3.0.2 “(The R Foundation for Statistical Computing, Vienna, Austria).

## Results

**Fig. 1:**
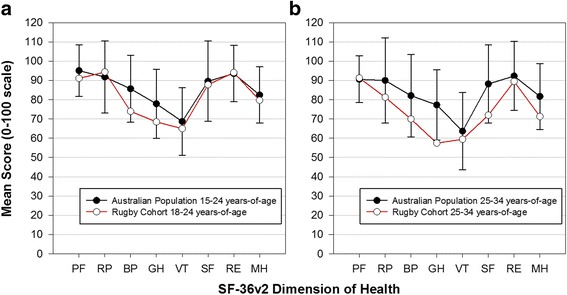
Comparison of mean health dimension score of amateur rugby players with age-matched Australian males. Legend: **a** Health comparison of rugby players 18–24 years-of-age with age-match Australian males (Mean [SD]) **b** Health comparison of rugby players 25–34 years-of-age with age-match Australian males (Mean [SD]). Dimensions of Health: PF physical function; RP role-physical; BP bodily pain; GH general health; VT vitality; SF social function; RE role-emotional; MH mental health

Data were collected from a cohort of 125 rugby players with a mean (SD) age of 24.3 (±4.9) years and 11.1 (±5.7) years of playing experience. Participants had mean (SD): SF-36v2 physical component score 47.2 (±9.8), SF-36v2 mental component score 50.0 (±9.3) and BMI 26.7 (±3.5). Participant characteristics as measured at baseline are listed in Table 1. The mean score of participants for each of the eight dimensions of health were generally within one standard-deviation of age-matched Australian males Fig. 1. Presented as supplementary material are the 0–100 and norm-based scores for the eight separate health domains of the SF-36v2 (Additional file 1: Tables S1 and S2).

**Table 1:** Mean (SD) baseline SF-36v2 physical and mental component scores and physical characteristics

	PCS	MCS	Height (cm)	Weight (kg)	BMI
Age group					
18–24	46.2 (10.2)	49.9 (10.3)	184.4 (6.6)	87.4 (10.6)	25.8 (3.2)
25–34	49.5 (8.4)	50.3 (7.0)	181.2 (7.3)	93.5 (11.6)	28.5 (3.3)
>35	46.0 (12.8)	46.7 (8.5)	186.8 (2.4)	103.0 (15.7)	29.4 (4.0)
Position					
Forward	46.9 (10.0)	50.3 (8.4)	184.1 (7.3)	92.8 (11.3)	27.4 (3.3)
Back	47.6 (9.6)	49.5 (10.4)	182.8 (6.3)	86.0 (10.5)	25.8 (3.5)
All					
	47.2 (9.8)	50.0 (9.3)	183.5 (6.8)	89.6 (11.5)	26.7 (3.5)

*PCS* physical component summary score, *MCS* mental component summary, *BMI* body mass index

A total of 129 injuries occurred during a combined period of 2465 match-hours of exposure. The overall IIR was 52.3 (95 % CI: 43.7–62.2) per 1000 match hours. Injuries were more frequently from trauma 46.7 (95 % CI: 38.5–56) than from overuse 5.7 (95 % CI: 3.1–9.5) The IIR for recurrent injuries was 3.2 (95 % CI: 1.4–6.9). Injury severity is presented in Table 2. The average injury severity was 9 (95 % CI: 7–12) days loss from play. Moderate and severe injuries (>1 week time-loss from play) comprised 36 % of all injuries. There were no fatal or catastrophic injuries.

**Table 2:** Frequency distribution of injury severity as measured by time loss from play (days of absence) due to injuries and injury rate per 1000 match-hours of exposure (IIR_MHE_)

Time loss from play	N (%)	IIR_MHE_ (95 % CI)
Less than one-week		
Slight (0–1 day)	55 (42.6 %)	22.3 (16.8–29.1)
Minimal (2–3 days)	4 (3.1 %)	1.6 (0.4–4.2)
Mild (4–7 days)	23 (17.8 %)	9.3 (5.9–14.0)
Greater than one-week		
Moderate (8–28 days)	26 (20.2 %)	10.6 (6.9–15.5)
Severe (>28 days)	20 (15.5 %)	8.1 (5.0–12.5)

Excluding the severity of one injury lost to follow-up

**Fig. 2:**
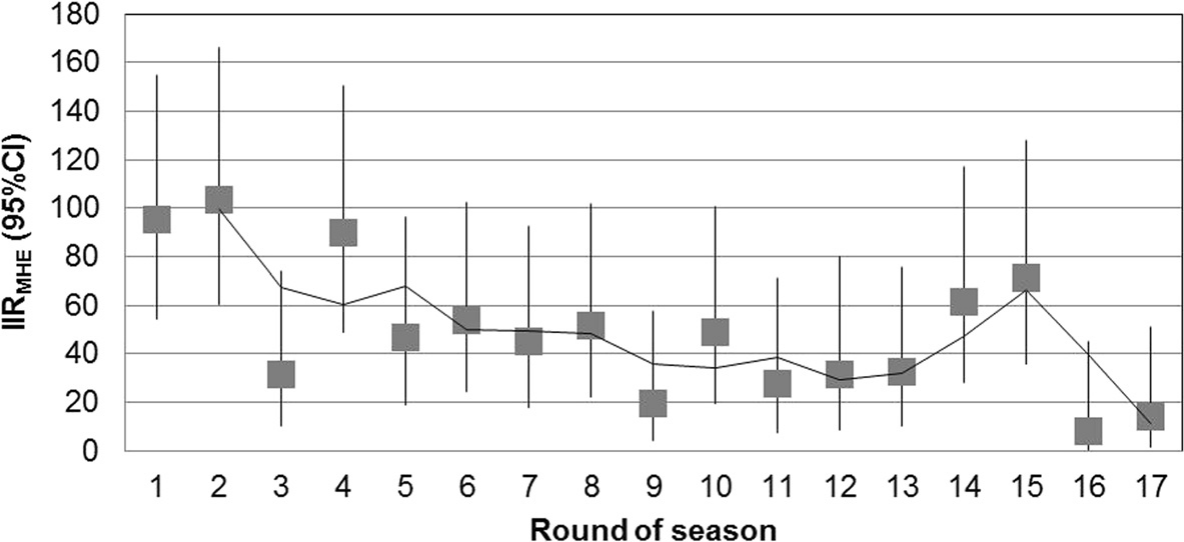
Injury incidence rates per 1000 match-hours of exposure (IIR_MHE_) with 95 % CI by round of season

The most common anatomical location of injury was the head and face (IIR 9.3 CI 5.9–14.0). Within the lower extremity, injuries at the knee were most frequent (IIR 7.3 CI 4.3–11.5); whereas the shoulder/clavicle was the most frequent injury location of upper extremity (IIR 7.3 CI 4.3–11.5). The most common injury types were ligament injuries (IIR 14.2 CI 9.9–19.7) followed by contusions (IIR 10.1 CI 6.6–15). Tackling was the most frequent mechanism of injury, followed by being tackled. The vast majority of injuries were not a result of dangerous play. The lowest number of injuries occurred in first grade; however, the difference between grades was not statistically significant. The proportion of injuries did not vary according to the time of match. Table 3 lists the injury frequencies by anatomical location, injury type, phase of play, grade of play, time of match and dangerous play. Injury incidence rates were similar across categories of age-group, position of play and body mass index (Table 4). Visual inspection of Fig. 2 suggests the IIR was highest in the first two rounds of the season (not further evaluated via statistical analysis).

**Table 3:** Frequency distribution of injuries by the anatomical location, type, phase of play, grade of play, time of match and dangerous play

	N	Percent % (95 % CI)
Anatomical location		
Head/face	23	17.8 (11.2–24.4)
Neck/cervical spine	6	4.7 (1.0–8.3)
Sternum/ribs/thorax	6	4.7 (1.0–8.3)
Abdomen	3	2.3 (0.0–4.9)
Low back	4	3.1 (0.1–6.1)
Sacrum/pelvis	1	0.8 (0.0–2.3)
Shoulder/clavicle	18	14 (8.0–19.9)
Upper arm	1	0.8 (0.0–2.3)
Elbow	4	3.1 (0.1–6.1)
Wrist	1	0.8 (0.0–2.3)
Hand/fingers/thumb	14	10.9 (5.5–16.2)
Hip/groin	2	1.6 (0.0–3.7)
Anterior thigh	2	1.6 (0.0–3.7)
Posterior thigh	8	6.2 (2.0–10.4)
Knee	18	14 (8.0–19.9)
Lower leg/achilles	6	4.7 (1.0–8.3)
Ankle	10	7.8 (3.1–12.4)
Other	2	1.6 (0.0–3.7)
Type of injury		
Ligament/sprain	35	27.1 % (19.5–34.8)
Hematoma/contusion/bruise	25	19.4 % (12.6–26.2)
Muscle	18	14 % (8.0–19.9)
Laceration	10	7.8 % (3.1–12.4)
Nerve	8	6.2 % (2.0–10.4)
Meniscus/cartilage/disc	7	5.4 % (1.5–9.3)
Concussion	6	4.7 % (1.8–3.0)
Other	6	4.7 % (1.0–8.3)
Dislocation/subluxation	4	3.1 % (0.1–6.1)
Tendon	4	3.1 % (0.1–6.1)
Fracture	3	2.3 % (0.0–4.9)
Abrasion	2	1.6 % (0.0–3.7)
Other bone	1	0.8 % (0.0–2.3)
Phase of play		
Tackling	44	34.6 (26.4–42.9)
Tackled	43	33.9 (25.6–42.1)
Ruck	13	10.2 (5.0–15.5)
Other	13	10.2 (25.6–15.5)
Collision	9	7.1 (2.6–11.5)
Maul	3	2.4 (0.0–5.0)
Scrum	2	1.6 (0.0–42.1)
Lineout	0	0 (0–0)
Grade of play		
1st grade	16	12.5 (6.8–18.2)
2nd grade	25	19.5 (12.7–26.4)
3rd grade	33	25.8 (18.2–33.4)
4th grade	29	22.7 (15.4–29.9)
Colts (under 19-years)	25	19.5 (12.7–26.4)
Time of match		
1st quarter	22	17.2 (10.7–23.7)
2nd quarter	39	30.5 (22.5–38.4)
3rd quarter	31	24.2 (16.8–31.6)
4th quarter	36	28.1 (20.3–35.9)
Dangerous play		
No	127	99.2 (97.7–100.7)
Yes	1	0.8 (0.0–2.3)

**Table 4:** Injury rate and rate ratios by age, position of play and BMI

	N	Percent (95 % CI)	IIR_MHE_ (95 % CI)	RR_MHE_ (95 % CI)
Age				
18–24	89	69 (61–77)	52.6 (42.2–64.7)	ref.
25–34	36	27.9 (20.2–35.6)	51.8 (36.3–71.7)	0.99 (0.67–1.45)
≥35	4	3.1 (0.1–6.1)	51.4 (14.0–131.6)	0.98 (0.37–2.56)
Year of participation				
≤10 years	79	61.2 (52.8–69.6)	52.8 (41.8–65.8)	ref.
>10 years	50	38.8 (30.4–47.2)	51.6 (38.3–68.0)	0.69–1.39
Position				
Forward	67	51.9 (43.3–60.6)	52.2 (40.5–66.3)	ref.
Backs	62	48.1 (39.4–56.7)	52.4 (40.2–67.2)	1.00 (0.71–1.42)
BMI				
<25	48	39.3 (30.7–48)	58.7 (43.3–77.9)	ref.
25 to 30	58	47.5 (38.7–56.4)	50.4 (38.3–65.1)	0.86 (0.59–1.25)
>30	16	13.1 (7.1–19.1)	40.0 (22.8–64.9)	0.68 (0.39–1.19)
SF-36v2 PCS				
NBS <47	56	43.4 (34.9–52)	54.7 (41.3–71.1)	1.05 (0.70–1.58)
NBS 47–53	40	31 (23–39)	51.9 (37.1–70.7)	ref.
NBS >53	33	25.6 (18.1–33.1)	49.1 (33.8–69.0)	0.95 (0.60–1.49)
SF-36v2 MCS				
NBS <47	38	29.5 (21.6–37.3)	53.7 (38.0–73.7)	0.93 (0.57–1.50)
NBS 47–53	28	21.7 (14.6–28.8)	57.9 (38.5–83.7)	ref.
NBS >53	63	48.8 (40.2–57.5)	49.5 (38.0–63.3)	0.85 (0.55–1.33)

*MHE* /1000 match-hours exposure, *BMI* body mass index, *PCS* physical component summary, *MCS* mental component summary, *NBS* norm-based score

Age, years of participation, BMI and SF-36v2 summary scores were treated as continuous variables in the Poisson mixed-effects generalised linear modelling. Multivariate modelling found fewer years of rugby participation, lower BMI and lower SF-36v2 mental component summary score was associated with higher IIR in amateur rugby. Whereas age, player position i.e., backs versus forwards and SF-36v2 physical component summary score were not associated with injury. Table 5 contains the results from the final Poisson mixed-effects generalised linear model.

**Table 5:** Rate ratio estimates per 1000 match hours of exposure (RR_MHE_) with 95 % CIs using Poisson mixed-effects generalised linear modelling

Study factor	RR_MHE_ (95 % CI)	P-value
Age	0.98 (0.95–1.02)	0.329
Years of participation	0.93 (0.88–0.99)	0.017*
Backs (ref. forwards)	0.96 (0.42–2.17)	0.920
BMI	0.97 (0.95–1.00)	0.020*
SF-36v2 PCS	1.00 (0.99–1.01)	0.682
SF-36v2 MCS	0.98 (0.97–1.00)	0.012*

*P < 0.05

## Discussion

Amateur RU players report similar pre-season health-related quality of life characteristics as the general population. During the competitive season, the match injury rate for amateur rugby players was 52.3 /1000 match-hours exposure, with the head location, ligament tissue type, and tackling mechanism being the most common. Approximately one-third of injuries resulted in >1 week of time-loss from play. Factors associated with higher injury rates in this study were fewer years of playing, lower BMI and lower mental health, but the relationships were weak.

The study used standardised injury definition and data collection procedures, which allows for comparison of our findings with similar studies. We were also able to maintain an individual player exposure log for a more accurate estimate of exposure adjusted injury rate, which is a common limitation of larger (multiple-club observations) studies conducted over several seasons. Due to logistical constraints our study’s hypothesis was explored with a cohort recruited from one amateur rugby club, followed over one-season and this limited sample size may have affected the precision of our estimates and the generalisability of our findings. Hence we likely identify moderate to strong associations. The notion that aspects of HRQoL may be associated with in-season sports injury is novel and adds to previous knowledge on the aetiology of sports injury in rugby.

To the best of our knowledge this is this first time multiple dimensions of HRQoL have been evaluated in a cohort of amateur rugby players. On average both physical and mental component summary scores were similar in this cohort of amateur rugby players when compared to age-matched Australian males. There were however some small divergences below the population norm score in the health dimensions of bodily pain and general health perception. Presumably these lower average scores in bodily pain and general health are linked to rugby related behaviours, such as physical contact in preseason training. Details about preseason injury status were not measured in this study, which limits us to speculation. Dimensions of health could also change with volume and type of rugby participation; consequently the temporal relationship between health, rugby exposure and injuries is an area for future research.

In this study, as expected, the overall match injury rate fell well below the high match injury rate of men’s international and level-1-club professional rugby (52.3 vs. 81.0 per 1000 match hours) [17]. However, the match injury rate in our cohort of amateur players was higher than level 2 professional rugby players (in Hong Kong and Japan) and a recent English cohort study of community rugby players (52.3 vs. 16.5–35.0 per 1000 match hours) [17, 26]. Also unexpected was the relatively low severity of injuries seen in this study (mean days of missed play 9 days); only around 16 % of injuries required more than one-month time loss from play. Results from a comparable study of community rugby players [26] report an average of 7.6 weeks missed per injury for all levels. The high proportion of slight injuries observed in our study may reflect a different risk profile in this cohort (i.e., a higher propensity for slight-mild injuries) or a greater sensitivity in our injury reporting.

Our description of match injuries was also similar to that seen in Scottish and English community rugby players in terms of location, mechanism, phase of play, player position and time of season [26, 27]. A point of difference from Roberts et al., [26] was that we found no difference in the rate of injury based on the time of the match or the grade of play. They, on the other hand, found injury incidence was lower in the first and second match quarters compared to the fourth and higher incidence in higher levels of competition. However, they observed a much larger sample and compared groups of clubs that play across wider levels of competitiveness, which likely accounts for the differences in injury rates across levels of play.

Previous studies have also measured rugby players’ height and mass preseason to assess the relationship between BMI and injury incidence [10, 11]. These studies suggested that players with BMIs higher than 25 kg/m^2^ are at greater risk of incurring an injury compared to players with BMIs less than 23 kg/m^2^, though these findings were not statistically significant. We observed the opposite finding, that is, players with higher BMIs were less likely to incur an injury; however, this finding was only significant when BMI was included as a continuous variable in a multivariate analysis. Similarly, player experience (the number of years of rugby participation) has been previously been evaluated as a predictive factor for injury occurrence in cohort studies [10, 11], but not found associated with injury rate. Unlike previous studies we did not categorise player experience and BMI in our model to avoid the known problems of loss of power and less precise estimation [28]. While our adjusted associations were significant the magnitude was small. At this time we believe inferences about the effects of BMI and experience on injury rate should be approached with caution, requiring further exploration in future research.

A simple yet commonly overlooked question in aetiological rugby studies is the impact of HRQoL of athletes on sport-related injury. The hypothesis for the current study follows work of Quarrie et al., [11] who, to the best of our knowledge, are the only group to have evaluated the potential role of a player’s preseason health and psychological wellbeing on injury. To provide a more comprehensive evaluation of rugby player’s overall health, HRQoL was measured with a robust measure that has been used with athletes [29, 30] and validated in patient populations [31]. A novel finding from our study was lower mental domain summary scores had a small association with higher rates of injury when controlling for other variables. Previous studies have found that rugby players who were injured in the previous season [12] or preseason [11] were more likely to be injured during the study season. It may be the case that previous injury adversely affects aspects of HRQoL such as physical functioning [29, 30]. Our model was established a priori with only a few potential predictors. A limitation of our study is that unaccounted for potential confounders such as previous injury may have distorted the prediction of HRQoL on injury incidence. Therefore further research is required to further evaluate the relationship between health and sports injury. Associations between health and rugby injury should be approached with caution at this time.

## Conclusions

In this one-season and one-club cohort study, amateur Australian rugby players were on average overweight and report similar HRQoL as other Australian men of the same age. Australian amateur rugby players have a higher rate of injury and lower injury severity than English community rugby players. However, the location, type, and mechanism of injuries align with previous reports in the rugby literature. When questioned prior to the commencement of the season, rugby players who have lower mental components of health, BMI and years of participation may have a slightly higher injury rate when baseline other factors are accounted for. However, these associations are weak and should be interpreted with caution if applied to preseason screening and prevention programs. Future research should include health among other factors to clarify the magnitude of injury risk associated with rugby.

## Additional file

Additional file 1: Table S1. SF-36v2 health domain scores: norm-based scores means and standard deviations. **Table S2.** SF-36v2 health domain scores: 0-100 means and standard deviations. (DOCX 21 kb)

## Abbreviations

95 % CI: 95 % confidence interval
BMI: body mass index
HRQoL: health-related quality of life
IIR: injury incidence rate
MHE: match-hour of exposure
OSICS-10.1: orchard sports injury classification 10 version 1
RR: rate ratio
SF-36v2: short form 36 item health survey version 2 standard
PCS: physical component summary
MCS: mental component summary
