# Supplementary material for: Match injuries in amateur Rugby Union: a prospective cohort study - FICS Biennial Symposium Second Prize Research Award
Source: Chiropr Man Therap. 2016 Jun 1;24:17. doi: 10.1186/s12998-016-0098-7 (PMC4888508; doi:10.1186/s12998-016-0098-7)
Supplement: Additional file 1: Table S1. — SF-36v2 health domain scores: norm-based scores means and standard deviations. Table S2. SF-36v2 health domain scores: 0-100 means and standard deviations. (DOCX 21 kb) [file 12998_2016_98_MOESM1_ESM.docx]

**Additional file 1**

**Table S1. SF-36v2 health domain scores: norm-based scores means and standard deviations**

|  |  | PF | RP | BP | GH | VT | SF | RE | MH |
| --- | --- | --- | --- | --- | --- | --- | --- | --- | --- |
| Age group | |  |  |  |  |  |  |  |  |
|  | 18-24 | 47.0 (17.8) | 51.4 (6.6) | 43.3 (14.7) | 44.7 (10.0) | 47.9 (10.1) | 49.1 (9.7) | 50.4 (10.4) | 48.1 (10.0) |
|  | 25-34 | 52.5 (10.4) | 50.4 (6.5) | 45.2 (15.5) | 45.6 (12.4) | 54.7 (7.1) | 51.4 (6.1) | 49.9 (8.6) | 48.6 (9.1) |
|  | >35 | 48.5 (8.3) | 46.3 (12.0) | 45.8 (17.7) | 42.2 (16.0) | 48.4 (11.6) | 43.5 (15.1) | 49.6 (6.7) | 45.1 (14.3) |
| Position |  |  |  |  |  |  |  |  |  |
|  | Forward | 48.1 (16.0) | 51.8 (5.7) | 44.3 (14.5) | 44.2 (10.9) | 49.0 (10.1) | 50.1 (8.5) | 51.0 (7.7) | 48.3 (9.2) |
|  | Back | 49.6 (15.5) | 49.9 (7.8) | 43.5 (15.6) | 45.8 (11.0) | 51.3 (9.2) | 49.2 (9.6) | 49.3 (11.6) | 48.0 (10.6) |
| All |  |  |  |  |  |  |  |  |  |
|  |  | 48.8 (15.8) | 50.9 (6.8) | 43.9 (14.9) | 44.9 (10.9) | 50.0 (9.8) | 49.7 (9.0) | 50.2 (9.7) | 48.2 (9.8) |

PF physical function; RP role-physical; BP bodily pain; GH general health; VT vitality; SF social function; RE role-emotional; MH mental health

**Table S2. SF-36v2 health domain scores: 0-100 means and standard deviations**

|  |  | | PF | RP | BP | GH | VT | SF | RE | MH |
| --- | --- | --- | --- | --- | --- | --- | --- | --- | --- | --- |
| Age group | |  |  |  |  |  |  |  |  |  |
|  | 18-24 | | 91.1 (23.9) | 94.4 (12.3) | 74.0 (25.5) | 68.5 (18.0) | 65.0 (17.7) | 87.8 (20.2) | 94.1 (15.2) | 79.7 (14.5) |
|  | 25-34 | | 93.6 (12.6) | 91.0 (14.5) | 71.7 (33.1) | 69.2 (22.5) | 73.1 (14.3) | 91.1 (12.5) | 92.1 (15.3) | 79.4 (15.6) |
|  | >35 | | 91.3 (8.5) | 81.3 (23.9) | 70.0 (35.6) | 57.5 (31.2) | 59.5 (23.7) | 72.0 (32.7) | 89.5 (12.6) | 71.3 (24.3) |
| Position | |  |  |  |  |  |  |  |  |  |
|  | Forward | | 91.3 (21.1) | 94.8 (11.8) | 73.5 (28.5) | 67.1 (19.8) | 65.6 (18.2) | 89.3 (17.8) | 94.7 (12.3) | 79.3 (14.6) |
|  | Back | | 92.6 (20.3) | 90.7 (15.2) | 72.8 (28.0) | 69.1 (19.8) | 69.4 (15.9) | 87.2 (20.0)0 | 91.8 (17.8) | 79.3 (15.8) |
| All | |  |  |  |  |  |  |  |  |  |
|  |  | | 91.9 (20.6) | 92.9 (13.6) | 73.2 (28.2) | 68.4 (19.8) | 67.4 (17.2) | 88.3 (18.8) | 93.4 (15.1) | 79.3 (15.1) |

PF physical function; RP role-physical; BP bodily pain; GH general health; VT vitality; SF social function; RE role-emotional; MH mental health
